# A new species of *Ophryotrocha* (Annelida, Eunicida, Dorvilleidae) from hydrothermal vents on the Southwest Indian Ridge

**DOI:** 10.3897/zookeys.687.13046

**Published:** 2017-08-01

**Authors:** Dong-sheng Zhang, Ya-dong Zhou, Chun-sheng Wang, Greg W. Rouse

**Affiliations:** 1 Laboratory of Marine Ecosystem and Biogeochemistry, Second Institute of Oceanography, State Oceanic Administration, Hangzhou, 310012, China; 2 State Key Laboratory of Satellite Ocean Environment Dynamics, Second Institute of Oceanography, State Oceanic Administration, Hangzhou, 310012, China; 3 Scripps Institution of Oceanography, UCSD, La Jolla, CA 92093-0202, USA

**Keywords:** Polychaeta, new species, systematics, hydrothermal vents, Indian Ocean

## Abstract

Dorvilleids were collected from hydrothermal vents on the Southwest Indian Ridge by manned submersible *Jiaolong*. These represent a new species of *Ophryotrocha* that is here described as *Ophryotrocha jiaolongi* **sp. n.** This is the first dorvilleid described from vents on the Southwest Indian Ridge. It most closely resembles another vent species, *Ophryotrocha akessoni* Blake, 1985 from the Galapagos Rift, but can be distinguished by its antennae, palps, jaw structure. The new species has particularly distinctive mandibles, which allow it to be easily identified.

## Introduction

*Ophryotrocha* Claparède & Mecznikow, 1869 is a diverse dorvilleid genus with more than 70 species described to date. These are distributed world-wide in diversified habitats from shallow water to deep-sea. A number of species in this genus are opportunistic or stress tolerant, can reach high abundance in reducing environments, such as hydrothermal vents and cold seeps, as well as whale and wood fall ecosystems (Desbruyères et al. 2006, Levin et al. 2003, Wiklund et al. 2009, Wiklund et al. 2012, Taboada et al. 2013, Salvo et al. 2014, Ravara et al. 2015). To date five *Ophryotrocha* species: *O. akessoni* Blake, 1985, *O. fabriae* Paxton & Morineaux, 2009, *O. globopalpata* Blake & Hilbig, 1990, *O. platykephale* Blake, 1985, and *O. wubaolingi* Miura, 1997 have been reported from hydrothermal vents (Blake 1985, Blake and Hilbig 1990, Miura 1997, Paxton and Morineaux 2009). These have been recovered in association with other animals such as siboglinid worms, mussels, clams, or in microbial mats (Desbruyères et al. 2006, Paxton and Morineaux 2009).

*Ophryotrocha* has previously been reported from vent fields on the Central Indian Ridge (Van Dover et al. 2001, Watanabe and Beedessee 2015) and the Southwest Indian Ridge (Copley et al. 2015). However, they have not been described. In this paper, dorvilleid worms from vents field on the Southwest Indian Ridge were studied and named as the sixth *Ophryotrocha* species from the hydrothermal vents.

## Material and methods

### Sample collection and morphological analyses

In January 2015, the China Ocean Mineral Resource R&D Association (COMRA) cruise DY35 was carried out by the research vessel *Xiangyanghong 9*, visiting the Southwest Indian Ocean. Sampling from the vents field was undertaken by the manned submersible *Jiaolong*. Specimens collected from two sites of the Longqi vent field, were sieved through a 250 μm mesh sieve, sorted, and preserved in 95% ethanol on board. The holotype and most paratypes are deposited in the repository of the Second Institute of Oceanography (RSIO), Hangzhou, China; additional paratypes are deposited in the Scripps Institution of Oceanography Benthic Invertebrate Collection (SIO-BIC), La Jolla California, USA.

Specimens were examined and photographed using a Zeiss V20 stereomicroscope with AxioCam ICc5 camera and a Leica DM5000 compound microscope. Jaws and chaetae were analyzed by scanning electron microscope (SEM). Jaws from both holotype and paratype were obtained after digesting anterior decapitated ends with a proteinase K solution at room temperature. Once the tissue was digested, the jaw elements were cleaned with distilled water and transferred to a glass cover slip. All elements for SEM were mounted on stubs and sputter coated with platinum-palladium and imaged using a Hitachi TM1000 scanning electron microscope.

DNA extraction was done with DNeasy blood and tissue kit (Qiagen, CA, USA) following the protocol supplied by the manufacturer. About 680 bp of CO1, 500 bp of 16S and 350 bp of H3 were amplified using primers LCO1490 and CO-E (Folmer et al. 1994, Bely and Wray, 2004) for CO1, 16SarL and 16SbrH (Palumbi 1996) for 16S and H3F and H3R (Colgan et al. 2000) for H3. PCR mixtures contained ddH_2_O, 1µl each primer (10 µM), 2 µl template DNA, 0.5 U of Taq polymerase (TAKARA, China), 2.5 µl of buffer solution (supplied by the polymerase manufacturer) and 0.5 µl of 2.5 mM dNTPs solution in a mixture of total 25µl. The temperature profile was as follows: 96°C/240s - (94°C/30s - 50°C/30s - 72°C/60s) * 35cycles - 72°C/420s. PCR products were purified with QIAquick PCR purification kit (Qiagen, CA, USA) following the protocol supplied by the manufacturer. Sequencing was performed by Sangon Biotech (Shanghai, China) on an ABI 3730XL DNA analyser (Applied Biosystems). Alignments of the three genes (CO1, 16S, H3) were performed using the program MAFFT (Katoh and Standley 2013) with all DNA data of dorvilleids available from Genbank. A maximum likelihood (ML) analysis was conducted by RAxML (Stamatakis 2014) using combined data of the three genes.

## Systematics

### Dorvilleidae Chamberlin, 1919

#### *Ophryotrocha* Claparède & Mecznikow, 1869

##### Ophryotrocha jiaolongi sp. n.

Taxon classificationAnimaliaAnnelidaDorvilleidae

http://zoobank.org/60CF9A6D-DBB9-4048-9C1C-501E94C580E0

Figs 1 , 2 , 3 , 4

###### Holotype.

(RSIO35301) Southwest Indian Ridge, Longqi vent field, HOV *Jiaolong* Dive 94, 49.6495°E; 37.7835°S, 2760m depth, 11 January 2015: ~ 10 mm long, 58 chaetigers; Paratypes: 21 specimens (RSIO35302) from same location as holotype; 7 specimens (RSIO35303) from Southwest Indian Ridge, Longqi vent field, 49.6501°E; 37.7836°S, 2737m depth; 8 specimens (SIO-BIC A6729) same locality as holotype.

###### Description.

**Figure 1.**
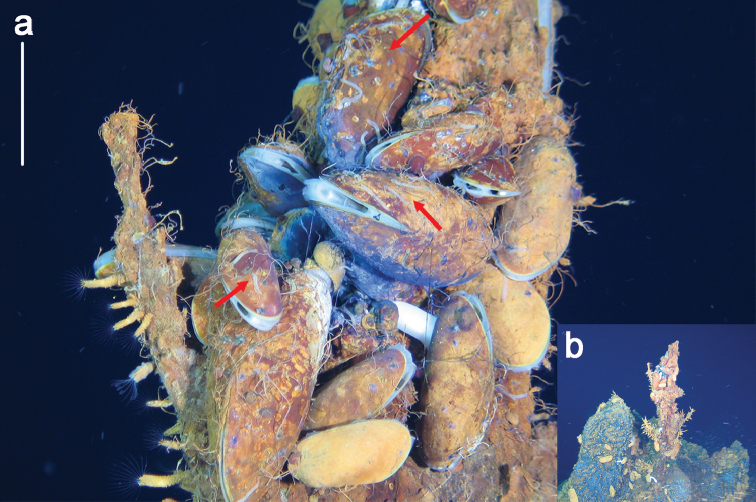
**a** *Ophyrotrocha jiaolongi* sp. n. (red arrows) specimens in vivo at the hydrothermal vent with mussels **b** same hydrothermal vent structure where *Ophryotrocha* specimens collected. Bars: 5 cm (**a**).

**Figure 2.**
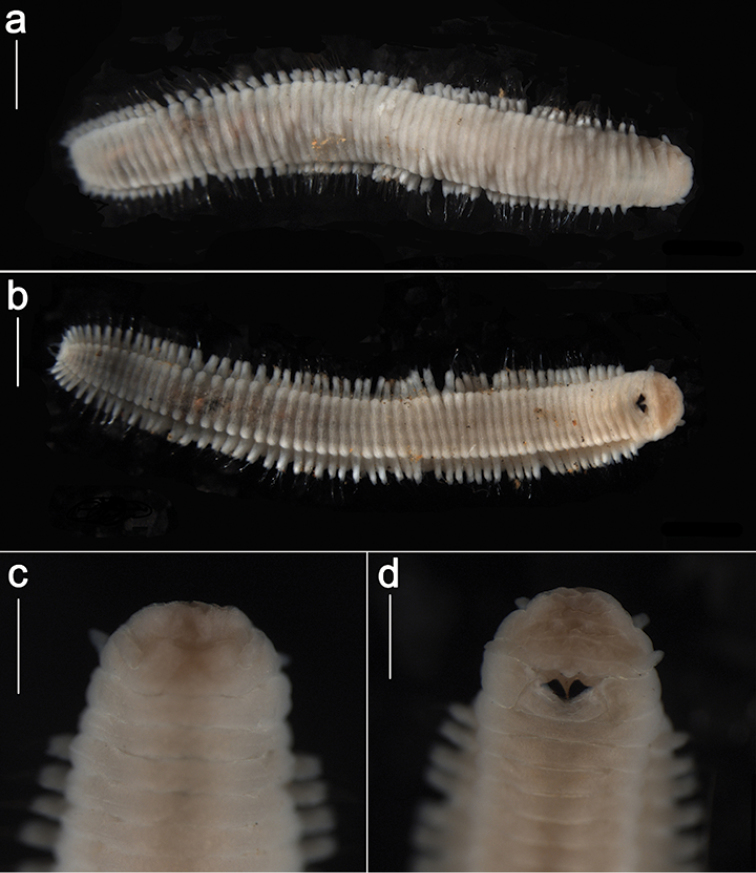
*Ophyrotrocha jiaolongi* sp. n., holotype. **a** dorsal view of whole body **b** ventral view of whole body **c** dorsal view of anterior region **d** ventral view of anterior region. Bars: 1 mm (**a, b**), 0.5 mm (**c, d**).

In life, body translucent (Fig. 1a), becoming opaque white after preservation (Fig. 2a, b). Body shape elongated, slightly dorsoventrally compressed, length up to 10 mm for more than 50 chaetigers, width 1.1 mm, uniform throughout the body, slightly tapering posteriorly (Fig. 2a, b). Prostomium wider than long, anterior margin rounded, posterior medial area slightly raised. Paired antennae short, digitiform, inserted dorsally, reaching to the anteriorly rounded edge of the prostomium (Fig. 2c, Fig. 4a). Paired palps digitiform, similar length as antennae, inserted ventral-laterally on prostomium (Fig. 2d). Eyes not visible. Peristomium with two rings sub-equal in length to following segments, the first ring with two notches ventrally on both sides of the jaw (Fig. 2d). Complete ciliary bands are observed on peristomium segments and chaetigers. Pygidium with terminal anus, two digitiform pygidial cirri inserted laterally, similar in length with the parapodia on the last chaetigers, a small median papilla ventrally placed (Fig. 2a, b, Fig. 4b).

**Figure 3.**
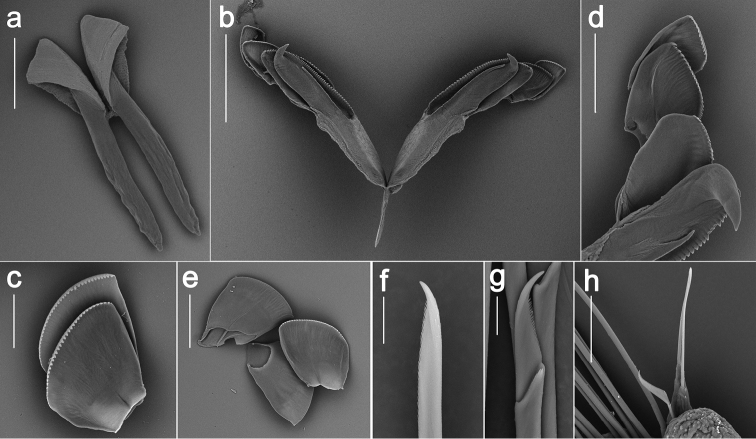
*Ophyrotrocha jiaolongi* sp. n. SEM images. (**a–c, f–h** holotype **d–e** paratype) **a** mandible, ventral view **b** forceps with free denticles 1-3 (D1-D3), dorsal view **c** free denticles 4-5 (D4-D5) **d** forcep with free denticles 1-3 (D1-D3) **e** free denticles 5-7 (D5-D7) **f** supra-acicular simple chaeta **g** sub-acicular compound chaeta **f** simple chaetae on sub-acicular lobe. Bars: 200 µm (**a, b**), 50 µm (**c, h**), 100 µm (**d, e**), 10 µm (**f, g**).

**Figure 4.**
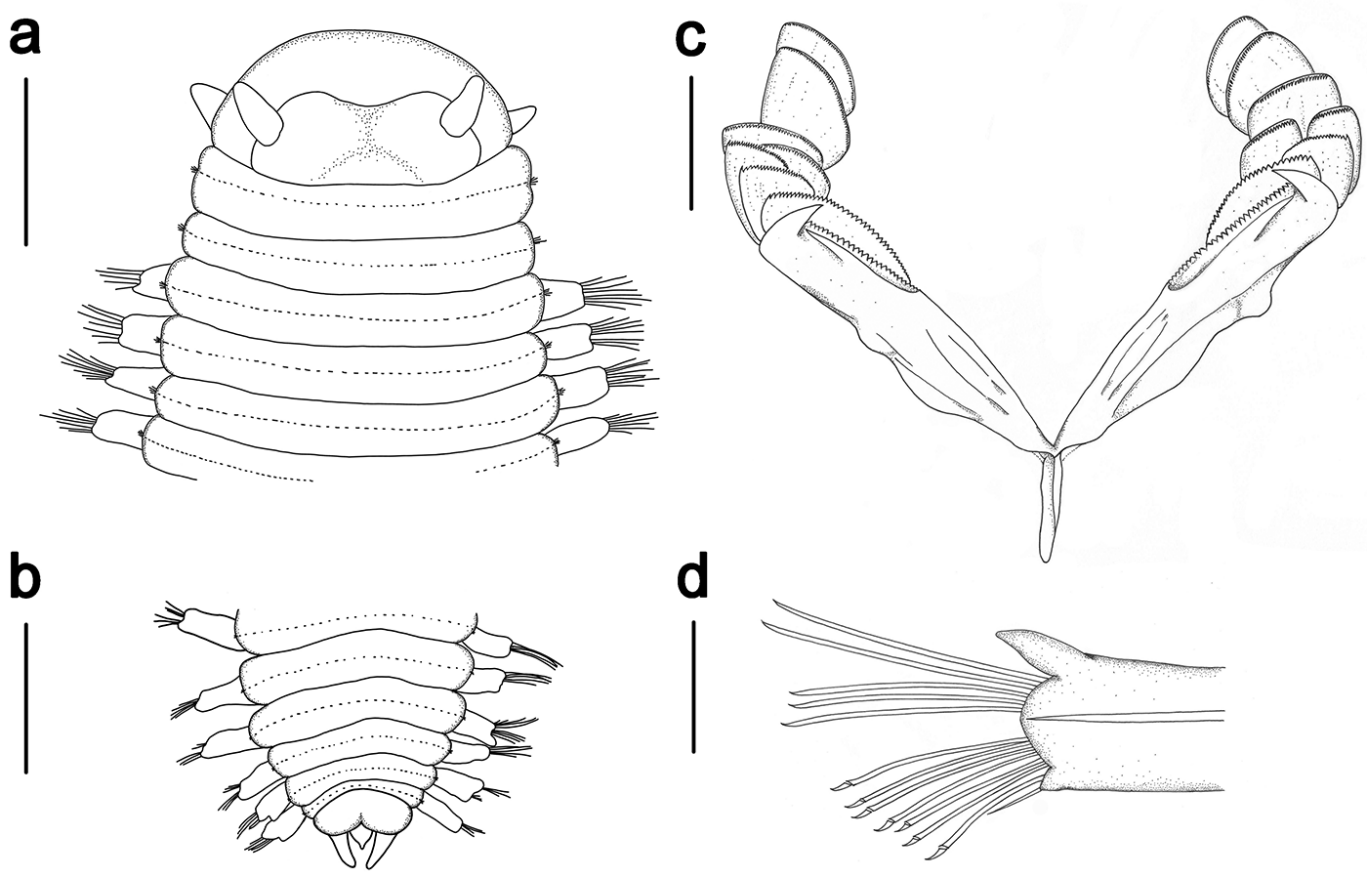
*Ophyrotrocha jiaolongi* sp. n. **a** prostomial, dorsal view **b** pydigium, dorsal view **c** maxillae with 7 pairs of free denticles **d** parapodia, anterior view. Bars: 500 µm (**a, b**), 200 µm (**c, d**).

Mandibles rod-like, each cutting plate composed of two sub-triangular plates, dorsal plate larger than ventral plate, fused together from anterior and middle sides, distal edge smooth, with single blunt peak, no serration or teeth observed (Fig. 3a). Maxillae P-type (Fig. 3b, Fig. 4c), forceps comb-like with more than 30 teeth slightly decreasing in size distally (Fig. 3b), seven pairs of free denticles (D), posteriormost pair (D1) oval shaped, longer than wide, smaller than forceps, D2-D7 shovel shape, wide sub-equal with long, except D3 clearly longer than wide (Fig. 3b, d). D2-D3 with a slightly larger main fang and similar long sharp teeth (Fig. 3b, d), D4–D5 with alternating long and short teeth (Fig. 3c), D6–D7 with serrated margin similar as D4–D5 or with smooth margin (Fig. 3e). K-type maxillae not found.

Parapodia uniramous, slightly broadening distally with long sub-conical dorsal cirri and short nub-like ventral cirri (Fig. 4d). Supra-acicular chaetae simple, distally serrated, tapering abruptly into a fang, bearing several tiny spines on both sides distally (Fig. 3f), maximum 8 chaetae per fascicle. Sub-acicular chaetae compound, blades with distally curved main fang and double row of spines, heterogomph shaft with several spines distally (Fig. 3g), maximum 11 chaetae per fascicle. Some parapodiau appear to have sub-acicular retractable lobes with 1–3 simple chaetae (Fig. 3h).

###### Etymology.

*Ophryotrocha jiaolongi* sp. n. is named after the Chinese manned submersible *Jiaolong*, in recognition of its successful expedition to the hydrothermal vents of the Southwest Indian Ridge.

###### Remarks.

The complex pharyngeal jaw apparatus, which is morphologically well characterized by the presence of ventral mandibles and dorsal maxillae, is an important diagnostic feature in Dorvilleidae (Rouse and Pleijel 2001). Mandibles of most *Ophryotrocha* species have been reported with a distally serrated edge or smooth anterior margin with anterior mandibular peaks. *Ophryotrocha jiaolongi* sp. n. has distinctive mandibles, with folded sub-triangular cutting plates, a distally smooth edge and a single anterior blunt peak, which easily distinguish it from other *Ophryotrocha* species. Among *Ophryotrocha* species, *O. jiaolongi* sp. n. most closely resembles *O. akessoni* Blake, 1985, in the general morphology of the prostomium, peristomium, ciliary bands, parapodia and chaetae, as well as in mandibular and maxillary structure. *Ophryotrocha jiaolongi* sp. n. differs from *O. akessoni* in having shorter antennae and palps and slight differences in jaw structure. The maxillae appear to be P-type in both species, although Blake referred to that of *O. akessoni* as tending towards K-type in the adult. *Ophryotrocha jiaolongi* has alternating large and small teeth on D4-D5, while *O. akessoni* has alternating large and small teeth on the forceps and D1.

###### DNA.

Sequences of *Ophryotrocha jiaolongi* sp. n. are deposited at NCBI Genbank with accession numbers CO1 KY906961-KY906965, 16S MF398963-MF398967, and H3 MF398968-MF398972. Preliminary phylogenetic analysis of the DNA data suggests that *O. jiaolongi* sp. n. is closely related to *O. clava* from whale bones. However, only one sequence of vent species (*O. globopalpata*) is currently available, which is located in a different clade from the new species. Further DNA data is being acquired from other vent *Ophryotrocha* species, which will help us to get a better understanding of the relationship among vents *Ophryotrocha* species in the near future (Zhang et al. in prep.).

**Figure 5.**
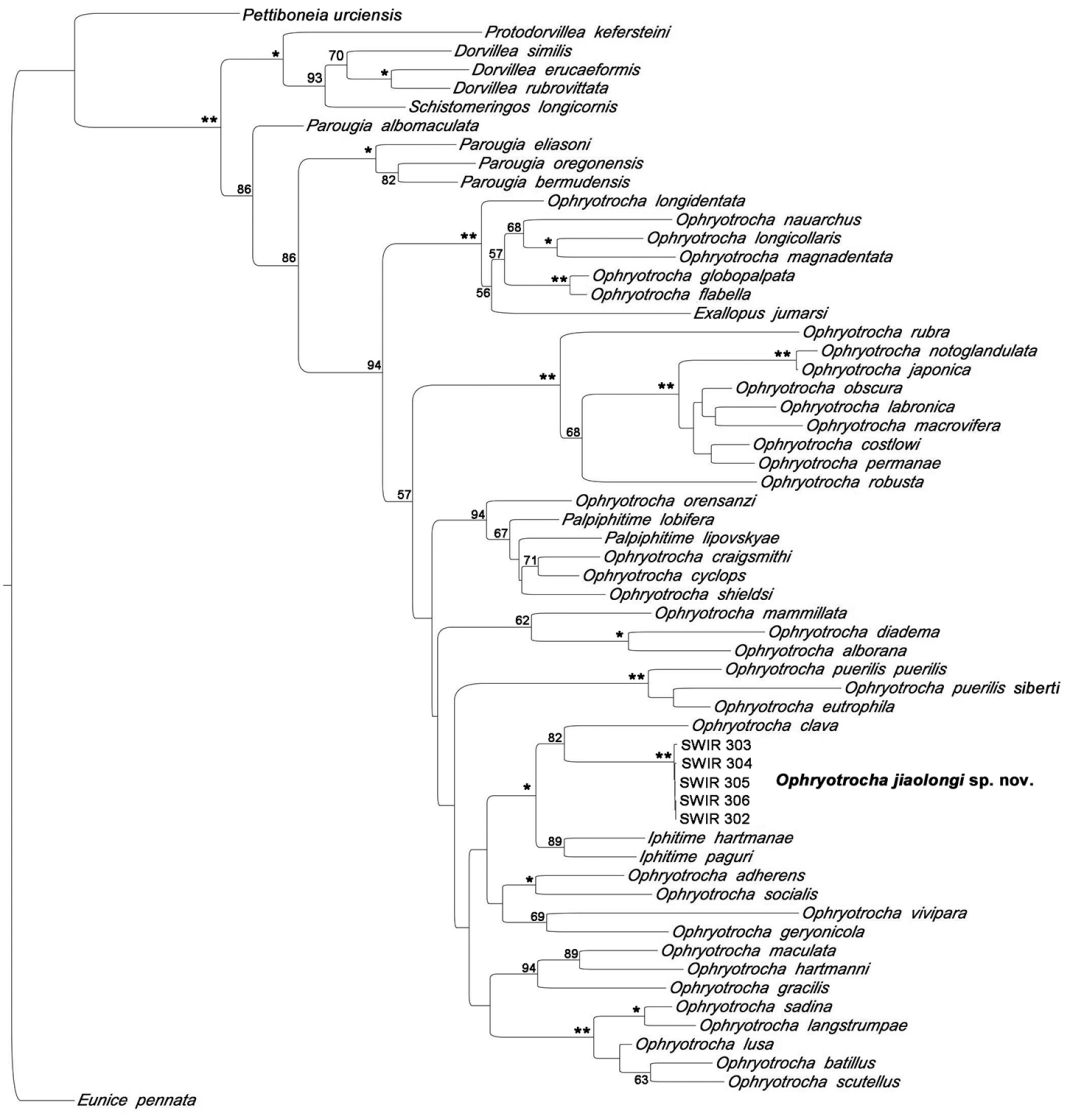
Maximum likelihood tree of the combined analysis from three genes (CO1, 16S, H3). Bootstrap support values (only higher than 50 were shown) were generated with a rapid bootstrapping algorithm for 1000 replicates. Double asterisk indicates support value of 100, single asterisk indicates support value of 95 or above.

## Supplementary Material

XML Treatment for Ophryotrocha jiaolongi
